# Multiple myeloma with *Echinococcus granulosus* infection diagnosed by detection of oligoclonal bands: A case report

**DOI:** 10.1097/MD.0000000000024709

**Published:** 2021-03-05

**Authors:** Jinghong Li, Xiaoyun Zhang, Hongjun Hao, Shiheng Fan, Yuming Xu

**Affiliations:** a Department of Neurology, First Affiliated Hospital of Zhengzhou University, Zhengzhou, Henan Province; b Department of Neurology, Neuroimmunology Laboratory, Peking University First Hospital, Beijing, China.

**Keywords:** *Echinococcus granulosus*, infection, isoelectric focusing electrophoresis, multiple myeloma, oligoclonal bands

## Abstract

**Rationale:**

Isoelectric focusing electrophoresis (IFE) is currently recognized as the gold standard for detecting oligoclonal bands (OCBs) in cerebrospinal fluid (CSF). To the best of our knowledge, however, no study has reported on type III OCBs using IFE. In this paper, we report on a rare case of multiple myeloma (MM) with *Echinococcus granulosus* infection diagnosed by IFE.

**Patient concerns:**

A 71-year-old man complained of weakness of the right lower extremity accompanied with fever (temperature range 37.8°C–38.2°C) for more than 6 months.

**Diagnoses:**

MM with *E granulosus* infection.

**Interventions:**

The IFE results identified a unique monoclonal band, indicating that the patient may have MM in conjunction with a distinct pathogen infection. He received anthelmintic treatment and bortezomib-thalidomide-dexamethasone therapy.

**Outcomes:**

The patient was followed up for 15 months. During that time, his temperature returned to normal, his *Medical Research Council Grading of Muscle Power* scale became 5, and his vital signs stabilized.

**Lessons:**

Detection of OCB type III indicated that the patient was diagnosed with MM accompanied by *E granulosus* infection. Thus, IFE of CSF may be an auxiliary diagnostic method for MM in the future.

## 1 Introduction

Isoelectric focusing electrophoresis (IFE) of cerebrospinal fluid (CSF) has important value in revealing the synthesis of immunoglobulin in the central nervous system (CNS). The appearance of oligoclonal bands (OCBs) in CSF is indicative of the occurrence of a humoral immune response in the CNS and is the most reliable indicator of immunoglobulin synthesis in the sheath of inflammatory demyelinating lesions of the CNS.^[1,2]^ The positive rate of OCBs in multiple sclerosis (MS) patients is 95%.^[3]^ There are 5 types of OCBs:

- type I, no bands in serum or CSF;
- type II, 2 or more bands in CSF but no bands in serum;
- type III, several bands in both serum and CSF, but more in CSF than in serum;
- type IV, same number of bands in serum and CSF, with mirror distribution; and
- type V, monoclonal bands in serum and CSF. Both type II and type III are recognized as OCB positive.^[4]^

OCB type III in this patient has unique characteristics and has not been reported yet. Comprehensively analyzing clinical data and valuable laboratory tests, the patient ultimately confirmed MM with *E granulosus* infection.

Echinococcosis, also known as hydatid disease, is caused by infection of *E granulosus* in the human body.^[5]^ Hepatic echinococcosis is the most common clinical echinococcosis, with the incidence of cerebral echinococcosis relatively low.^[6]^

## 2 Case presentation

A 71-year-old man experienced weakness of the right lower extremity accompanied by fever (temperature range 37.8°C–38.2°C) for more than 6 months, during which time he received treatment in local community hospitals. He had no contact history with an epidemic area. He was admitted to our hospital (First Affiliated Hospital of Zhengzhou University, China) when his symptoms relapsed. This study was approved by the Ethics Committee of the First Affiliated Hospital of Zhengzhou University, China (Ethical code: 2020-KY-339), and informed consent was obtained from the patient.

On admission, neurological examination of the patient did not show any apparent abnormalities. Electrolytes, coagulation function, and brain natriuretic peptides were normal. Tumor markers and tuberculosis-related tests in serum were negative, as were the results of relative examination of connective tissue disease. Dynamic electrocardiogram of the heart showed no significant changes, and the electroencephalogram was normal. Antibodies against neuronal cell-surface antigens (NMDAR, AMPAR, CASPR2, GABA1, GABA2, and LGI1) and onconeuronal antibodies (Hu, Yo, Ri, CV2, amphiphysin, and PNMA2) were all negative in serum and CSF. However, leukocytes were increased, glucose was significantly decreased, and eosinophils and plasma cells were found in the CSF cytology (Table 1). Routine blood tests also showed that the patient had mild anemia. Three consecutive erythrocyte sedimentation rates (97 mm/h, 106 mm/h, and 132 mm/h, respectively) indicated an abnormal increase (normal range: 0–15 mm/h).

**Table 1:** Three cytological results of CSF during hospitalization in 2019.

Objective	First	Second	Third	Normal range
White cell (×10^6^/L)	130	64	104	0–5
Red cell (×10^6^/L)	2	2	4	0
Lymphocyte ratio (%)	54	74	56	60-70
Monocyte ratio (%)	21	17	22	30-40
Activated monocyte ratio (%)	4	2	2	0–4
Neutrophil ratio (%)	5	2	5	0
Eosinophil ratio (%)	13	4	5	0
Plasma cell ratio (%)	1	1	0	0
Protein (mg/L)	762.5	710.7	690.3	150-450
Glucose (mmol/L)	0.92	0.56	0.46	2.5–4.5
Chloride (mmol/L)	125.4	112.2	131.7	120-130

**Figure 1:**
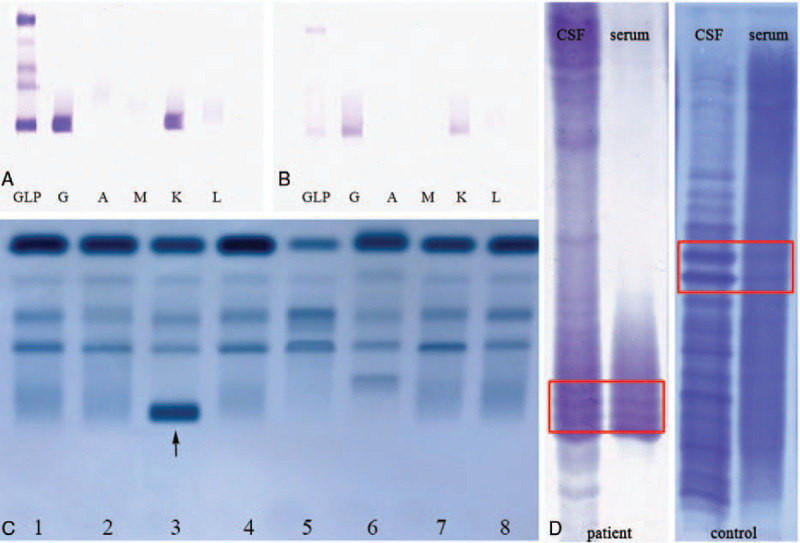
A. Serum immunofixation electrophoresis, IgG/Kappa type; B. Cerebrospinal fluid immunofixation electrophoresis, IgG/Kappa type; C. Serum protein electrophoresis. Arrow indicates patient outcome, with abnormal proteins; D. Oligoclonal band in cerebrospinal fluid of present case (left) and common type III of control (right). Red box shows oligoclonal bands. CSF = cerebrospinal fluid.

The results of immunofixation electrophoresis of serum (Fig. 1A) and CSF (Fig. 1B) and serum protein electrophoresis (Fig. 1C) were all IgG/Kappa type. The OCB was type III, with the monoclonal band exhibiting unique characteristics (Fig. 1D).

**Figure 2:**
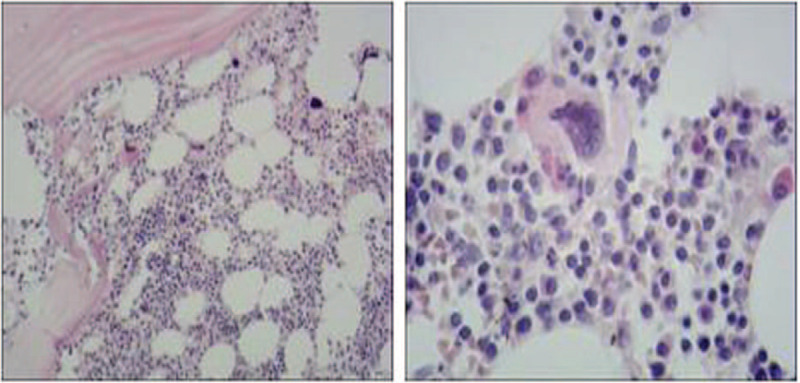
Bone marrow biopsy showing abnormal plasma cell increase (30%–40%) with focal distribution.

**Figure 3:**
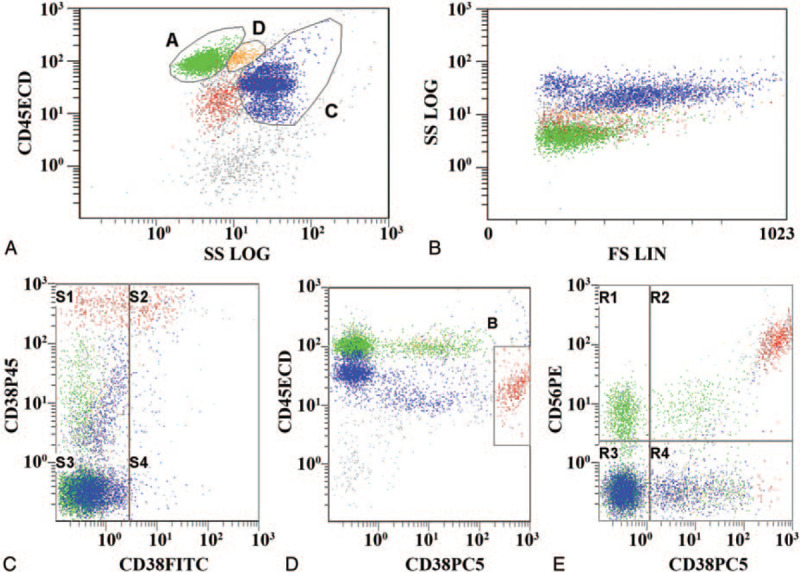
Flow cytometric analysis of leukemia in bone marrow. CD138+ CD38str+ plasma cells accounted for 6.0% of nuclear cells and were located in the CD45-positive region, with high expression of CD138, CD38str, CD81, CD56, and cKappa, suggesting abnormal clonal plasma cells.

Bone marrow biopsy showed an abnormal plasma cell increase (30%–40%) with focal distribution. (Fig. 2). For whole blood flow cytometry: the CD138+ CD38str+ plasma cells accounted for 6.0% of the nuclear cells, and were located in the CD45-positive area, with high expression of CD138, CD38str, CD81, CD56, and cKappa, and no expression of CD27, CD20, CD10, CD19, CD33, CD117, CD25, CD28, and cLambda, thus indicating abnormal clonal plasma cells (Fig. 3).

Immunohistochemical results of bone marrow tissue showed CD38+, CD138+, κ+, λ-, CD56+, CD20-, CD3-, Mum1+, suggesting plasma cell myeloma, consistent with the flow cytometry results.

**Figure 4:**
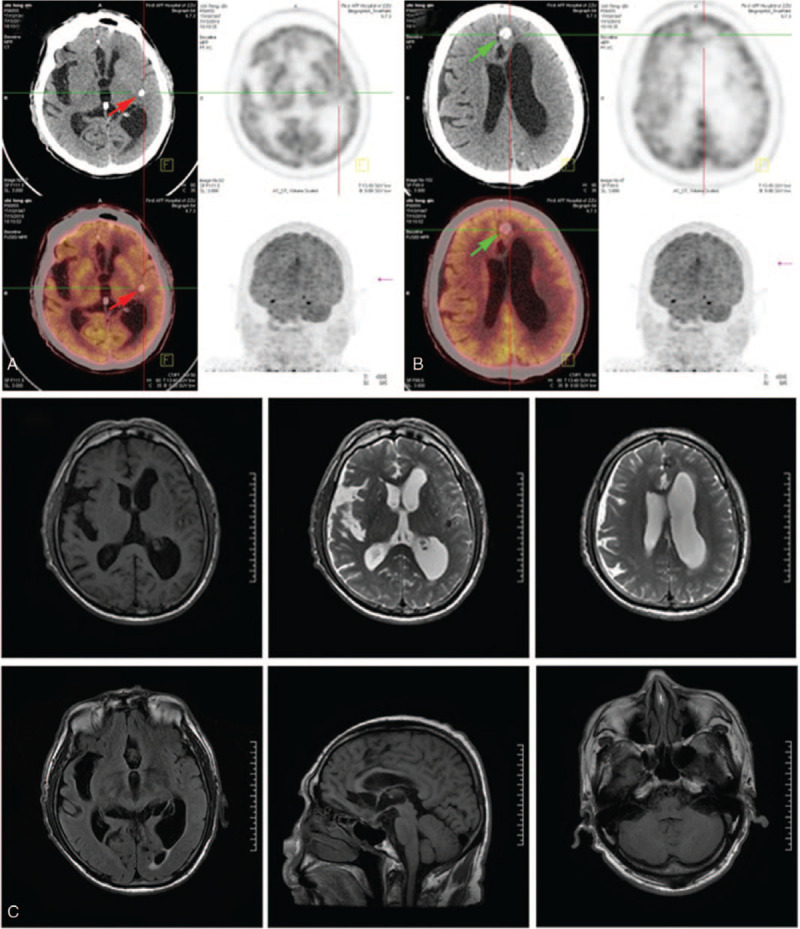
A. Positron emission tomography-computed tomography of head. Red arrow on left indicates a high-density shadow at basal ganglia; B. Green arrow in frontal lobe indicates a high-density shadow, considered calcification from cysticercosis. C. Magnetic resonance imaging (MRI) of head. Anterior longitudinal fissure, suprasellar cisterna, anterior pontine cisterna, multiple abnormal signals in left hippocampus, imaging features of cysticercosis.

Positron emission tomography-computed tomography showed that the metabolism of the central bone marrow was active, consistent with the features of myeloma (Fig. 4A and B). Head magnetic resonance imaging (MRI) suggested the presence of a calcification (Fig. 4C), indicating a potential cysticercoid, which was not detected via ophthalmologic examination. However, CSF and serum tests were positive for cysticercoid and hydatid antibodies. Additionally, 137 sequences of *E granuloses* were detected by next-generation sequencing (NGS) of whole blood. Based on the above results, it was concluded that the patient was diagnosed with MM, accompanied by a parasite infection.

His fever was treated with cefminox sodium and biapenem. The deworming treatment lasted for a month, and he also accepted chemotherapy, immunoregulation, and other treatments for MM. The patient was followed up for 15 months, during which time his temperature returned to normal, his muscle tone became 5 and his vital signs stabilized. Head MRI showed no significant changes.

## 3 Discussion

OCBs refer to the formation of several narrow discontinuous bands in the γ-globulin region during electrophoresis,^[4]^ with 5 types defined, as described above. In the current case, the OCBs were identified as type III. The bands in the red boxes in Figure 1D were considered to be monoclonal bands. Compared with the healthy volunteer control, the patient bands exhibited unique characteristics. Furthermore, the monoclonal bands were found in both serum and CSF, though additional bands were detected in the CSF of the patient (Fig. 1D), implying blood-brain barrier damage and IgG intrathecal synthesis. These results indicated that antibodies were not produced by the CNS, but that abnormal immunoglobulin synthesized by plasmacytoma entered the CNS through the blood-brain barrier damage. This pattern suggested a special pathogen infection, which was subsequently identified by NGS. Eosinophilia was found in CSF cytology, also indicating that the patient was infected by a parasite, consistent with the NGS results. We performed CSF and serum immunoelectrophoresis, serum protein electrophoresis, and urine Bence-Jones protein electrophoresis, with results indicative of IgG/Kappa type. Subsequent bone marrow smear, bone marrow biopsy, and immunohistochemical analysis were strongly suggestive of MM. The bone marrow biopsy found atypical plasma cells (Fig. 2). Leukemia immunotype by flow cytometer indicated that the patient was suffering from myeloma (Fig. 3). Based on the above findings, the patient was ultimately diagnosed as having MM. In this case, monoclonal bands were simultaneously found in the OCBs of serum and CSF, indicating the existence of monoclonal proliferative accessory protein.^[7]^ Type III OCBs are often found in inflammatory demyelination of the CNS caused by autoimmune diseases. In this case, the antibodies of autoimmune encephalitis and paraneoplastic syndrome were negative in serum and CSF, and the electroencephalogram was normal, suggesting that neuroautoimmune diseases could be excluded.

MM is a malignant monoclonal B cell disease, which shows frequent occurrence in the elderly.^[8]^ In recent years, the incidence of MM has seen an increase. The characteristics of MM include abnormal proliferation of plasma cells in the bone marrow accompanied by the over production of monoclonal immunoglobulins or light chains.^[9,10]^ MM is often misdiagnosed because of its insidious onset and lack of specificity. Bardwick et al indicated that the affinity of plasma cell clones to human peripheral nerve myelin sheaths is related to the pathogenesis of the disease.^[11]^ In the current case, the patient's main symptoms were weakness of the right lower limb accompanied by a fever for half a year. We speculated that pathogenesis was related to plasma cell disease, i.e., toxic substances secreted by plasma cells impacted organs of the whole body, resulting in multiple system damage.

In this reported case, eosinophils were increased in CSF cytology (Table 1). Thus, we performed parasite detection by enzyme-linked immunosorbent assay in CSF and serum, with IgG antibody-positive results obtained for both cysticercoid and hydatid. Both MRI and positron emission tomography-computed tomography images of the patient's brain showed multiple lesions, which were considered as cerebral cysticercosis (calcified) (Fig. 4). To further clarify the diagnosis, NGS was performed, with 137 hydatid sequences but no cysticercoid sequences found. NGS detects free nucleic acids, which persists short time in vivo. Sequencing results indicated that the patient had present infection.^[12,13]^ Thus, the above results suggested that the patient had recent hydatid infection and early cysticercosis. No hydatid features were found in the head MRI of the patient. This was likely related to the long incubation period of hydatid disease and difficultly in diagnosing early infection by imaging examination.^[14]^

Parasitic infection and MM are 2 clinically unrelated diseases, and cases showing both are rare. As such, this case could be easily misdiagnosed because of its rarity and particularity. The reasons are as follows:

(1) The clinical manifestations of MM are complex and diverse due to the different degrees of malignant hyperplasia and infiltration ranges of MM, different numbers and types of monoclonal immunoglobulins, and lack of specificity in clinical symptoms. In this case, limb weakness was the initial symptom.

(2) MM is occult but can cause multiple lesions. In clinical practice, we often pay attention to the lesions of a single system but fail to analyze the symptoms of multiple systems. In the current case, the patient's condition was complex, and the above symptoms could be explained by the primary disease. Without the identification of distinct OCBs and application of years of clinical experience, it can be difficult to diagnose MM.

(3) According to imaging and CSF cytology, the patient was diagnosed with a parasite infection and received appropriate treatment. After subsequent examination, a unique OCB was identified, and further experimental examinations were carried out. Finally, the patient was diagnosed with MM.

## 4 Conclusions

To the best of our knowledge, this study is the first to report on a case of OCB type III-related myeloma, which presented as a motor disturbance in the right lower extremity. The OCB findings indicated type III with *E granuloses* infection and MM. Thus, IFE of CSF could be applied as an auxiliary diagnostic method for MM in the future.

## Author contributions

**Methodology:** Jinghong Li, Hongjun Hao, Yuming Xu.

**Validation:** Xiaoyun Zhang, Shiheng Fan.

**Writing – original draft:** Jinghong Li, Hongjun Hao.

**Writing – review & editing:** Jinghong Li, Xiaoyun Zhang.

**Investigation:** yuming Xu.

**Project administration:** yuming Xu.

**Software:** Shiheng Fan.

**Supervision:** hongjun Hao.

**Validation:** hongjun Hao.

**Visualization:** xiaoyun Zhang, hongjun Hao.

**Writing – original draft:** Jinghong Li.

**Writing – review & editing:** xiaoyun Zhang, Shiheng Fan.
